# Knowledge, attitudes, and practices of migraine management among primary health care physicians in Al-Madinah Al-Munawarah

**DOI:** 10.25122/jml-2024-0350

**Published:** 2024-11

**Authors:** Khalid Wasel Al-Quliti, Abdulelah Nawaf Alraddadi, Abdulmajeed Waleed Alnoaman, Mohammed Abdullah Alahmadi, Zakaria Yahya Khawaji, Waleed Khalid Alquliti, Sultan Abdulaziz Aljohani

**Affiliations:** 1 Department of Medicine, College of Medicine, Taibah University, Madinah, Kingdom of Saudi Arabia.; 2 College of Medicine and Surgery, Taibah University, Madinah, Kingdom of Saudi Arabia

**Keywords:** Migraine, headache disorders, physicians, primary care, Saudi Arabia

## Abstract

Migraine is a burdensome primary headache disorder with a global prevalence ranging from 15-18%. Our study aimed to assess the knowledge among primary healthcare physicians regarding migraine and evaluate whether their management practices align with current advances. This descriptive cross-sectional study included 212 primary healthcare physicians working in Al-Madinah Al-Munawarah, Kingdom of Saudi Arabia. Data were collected using a self-administered, validated questionnaire distributed at clinics, with participant consent, to ensure privacy. A total of 212 responses were collected, the majority were from men (53.8%) and participants less than 30 years old (43.9%). Most participants held a Bachelor of Medicine, Bachelor of Surgery (MBBS) qualification (general practitioners), accounting for 56.1% of the sample. The results revealed that 83.5% had a high level of knowledge about the diagnostic criteria for migraine. Factors associated with a higher level of knowledge were female gender and age group less than 30 years. However, most participants (62.3%) were not familiar with the new acute and preventive migraine treatments. The findings of this study indicate good knowledge, attitude, and practicing habits among our participants. However, there were clear deficiencies in understanding the latest advancements in migraine treatment. We recommend implementing continuous education programs regarding the advances in migraine treatment among primary care physicians in Al-Madinah Al-Munawarah.

## INTRODUCTION

Migraine is a burdensome primary headache disorder defined clinically according to the third edition of the International Classification of Headache Disorders (ICHD-3) as a unilateral throbbing headache that lasts for 4–72 hours and is accompanied by nausea, vomiting, photophobia, and/or phonophobia. It is also described as a syndrome characterized by chronic or recurrent attacks of headache associated with other neurological manifestations such as transient motor and somatosensory impairment [1-5].

The global prevalence ranges from 15 to 18%, corresponding to over 1 billion people who experience migraine, making it the second most prevailing neurological disorder. The prevalence peaks between the ages of 35 and 39, with a female-to-male ratio of 3:1 [4,6,7].

In large-scale studies from Saudi Arabia, the prevalence of migraine was reported between 26.97% and 28.7%, with a female predominance (female-to-male ratio of 2.9:1), which is consistent with the global ratio. The most affected age group was between 18 and 25 years old [8,9]. Some patients with migraine may have other associated comorbidities such as sleep disorders, depression, stroke, fibromyalgia, and myocardial infarction. These comorbidities significantly impact the individual's personal, social, academic, and occupational life, thereby impairing their health-related quality of life (QoL). Globally, migraine has been identified as the second most disabling disease, following low back pain [10].

Migraine could be classified into migraine with aura, migraine without aura, chronic migraine, and probable migraine. The diagnosis is clinical and based on the third edition of the ICHD-3 [5,11]. For acute migraine attacks, the first-line pharmacotherapy consists of over-the-counter non-steroidal anti-inflammatory drugs (NSAIDs). If these are insufficient, triptans are considered the second-line treatment, followed by third-line options, including ditans and gepants. In the preventive treatment of chronic migraine, beta-blockers and topiramate are recommended as first-line agents. Second-line prophylactic options include flunarizine and amitriptyline. Calcitonin gene-related peptide (CGRP) monoclonal antibodies serve as the last-line prophylactic therapy for patients who do not respond to other treatments [2].

Several studies worldwide have assessed the knowledge and practices of primary healthcare physicians regarding migraines. For example, a regional study in Turkey evaluated the awareness of primary healthcare physicians toward migraine and found that only 10.5% of participants could accurately provide the complete diagnostic criteria for migraine without aura. This study emphasized the need for educational programs targeting primary care physicians to improve the management of migraines [12]. Another study in Italy aimed to assess the knowledge, attitude, and practice of Italian occupational physicians (OP) regarding migraines. The results demonstrated that participating OP had an excellent understanding of migraine and their associated triggers. However, some persistent false beliefs and common misunderstandings impaired the proper management of migraine [13].

Regarding the management options of migraine, a study done in India among 200 primary health care physicians revealed that 70% of participants prescribed pharmacological treatment, 25% focused on non-pharmacological treatment, while the remaining 5% stated that they usually refer migraine patients to a neurologist [14]. Another study in Burkina Faso found that participating general practitioners had good knowledge regarding the diagnostic criteria and the acute treatment of migraine. However, the participants did not know about preventive treatment of migraines [15]. Locally, a study conducted in Jeddah, Saudi Arabia, assessed general practitioners' knowledge of the diagnosis and management of chronic migraine headaches. The findings indicated their knowledge was inadequate, which could significantly impact referral patterns to secondary healthcare facilities and affect the overall quality of migraine care [16].

The Saudi for Evidence-Based Health Care (EBHC) released its executive recommendation summary in 2016, providing the latest institutional guidelines for migraine management in Saudi Arabia. For acute management, the panel recommended NSAIDs or metoclopramide as the first-line abortive therapy. Beta-blockers, valproate, or topiramate were suggested as the preferred preventive regimens for prophylactic treatment. However, the guidelines did not recommend any novel therapeutic agents [17].

Given the high burden of migraine in the Kingdom of Saudi Arabia, as discussed earlier, and the lack of data on this topic in Al-Madinah Al-Munawarah, this study addressed a critical knowledge gap. To date, no research has assessed general practitioners’ knowledge, attitudes, and practices regarding migraine management in primary healthcare settings in this region. This study seeks to provide valuable insights into migraine care and identify areas for improvement among primary healthcare physicians in Al-Madinah Al-Munawarah.

The objectives of this study were:

1. To determine the level of knowledge about migraine among primary health care physicians in Al-Madinah Al-Munawarah.
2. To assess the attitudes of primary health care physicians towards migraine management in Al-Madinah Al-Munawarah.
3. To assess the practice of migraine management among primary health care physicians in Al-Madinah Al-Munawarah.
4. To determine the factors that might affect the knowledge, attitude, and practice of migraine management among primary health care physicians in Al-Madinah Al-Munawarah.

## MATERIAL AND METHODS

### Study design and setting

This descriptive cross-sectional study was conducted from December 2022 to January 2024 to assess primary healthcare physicians’ knowledge, attitudes, and practices regarding migraine management in Al-Madinah Al-Munawarah, Kingdom of Saudi Arabia. Data were collected using a self-administered questionnaire distributed to participants at their clinics.

### Sample size and recruitment

The inclusion criteria were primary healthcare physicians in Al-Madinah Al-Munawarah who agreed to participate and complete the questionnaire. Participants who refused to participate or had incomplete questionnaires were excluded. According to a recent study conducted in the region, there were 269 primary healthcare physicians in Al-Madinah Al-Munawarah [18]. The required representative sample with a margin of error of 5% and a confidence level of 95% was 159, as determined by the open-source calculator OpenEpi website, version 3.01 [19].

To ensure balanced representation across the city, we divided the study area into four regions (North, East, South, and West). In each region, 10–13 primary healthcare centers were identified, and 40 or more participants were included, with 3–4 physicians sampled from each center. A convenience sampling technique was used to ensure fair representation, particularly from smaller or peripheral centers.

### Instruments and data collection

The data was collected using a previously validated self-administered questionnaire to assess the knowledge, attitude, and management of migraines among primary healthcare physicians in Burkina Faso [15]. We made some minor modifications to the questionnaire to make it more suitable for our research objectives. Primary health care physicians in the clinics completed the questionnaire using digital tablets provided by the research team to ensure completeness, accuracy, and actual representation of the responses. The questionnaire was provided online, and access to the form was granted to the research team via their tablets to avoid unintended responses. The research team did not intervene in the process; they only explained the purpose of the research and obtained consent before the start of the questionnaire. The questionnaire consisted of five parts. The first part consisted of informed consent and the participant’s agreement to participate in the study. The second part consisted of six questions to address the participant’s personal and sociodemographic information (age, gender, academic qualifications, professional experience, whether or not the participant completed an internship in neurology, and personal history of migraine headaches). The third section of the questionnaire focused on assessing participants' knowledge of migraine diagnostic criteria, specifically those outlined in the ICHD-3 [11]. Participants were presented with 14 diagnostic features to minimize bias: seven correct features representing classical diagnostic criteria for migraine and seven incorrect or atypical features. This approach aimed to accurately assess participants' true knowledge while reducing the likelihood of guesswork or misunderstanding. Participants were asked to classify each feature as 'yes', 'no', or 'I don’t know'. Knowledge levels were then categorized based on the number of correct responses: low (0–2 correct features), intermediate (3–5 correct features), or high (6–7 correct features). The fourth section of the questionnaire focused on assessing participants' attitudes toward the burden, diagnosis, and management of migraine. Participants were presented with seven positive and negative statements and asked to indicate their level of agreement using a five-point Likert scale: strongly agree, agree, neutral, disagree, or strongly disagree. The fifth section evaluated participants' practices in managing migraines. It comprised 12 multiple-choice questions addressing various management process components, including diagnostic investigations, acute attack treatments, prophylactic treatments, awareness of new therapeutic options for acute and preventive care, and referral patterns.

Surveys were administered in person, and participants were observed during completion to ensure that responses were authentic and not influenced by external sources, such as consulting textbooks or online resources.

### Data analysis

After data collection, responses were organized, checked for completeness, and entered into Microsoft Excel. Confidentiality was achieved by not using any names or identifiable data that could lead to the exposure of the participant’s identity. The collected data was sorted, coded, secured by password protection, and preserved safely. Access to the data was available only to the researchers. The data were then transferred to the Statistical Package for the Social Sciences (SPSS) for analysis. Continuous variables, such as the knowledge score (out of 7), were summarized using means and standard deviations, while categorical variables were reported as frequencies and percentages. Parametric statistical tests were applied to compare means: *t*-tests for comparisons between two groups and one-way analysis of variance (ANOVA) for comparisons among more than two groups. A *P* value of less than 0.05 was considered statistically significant.

## RESULTS

The socio-demographic and professional data of the participants are presented in Table 1. This study included 212 primary health care physicians from governmental primary health care centers across the four regions of Al-Madinah. The majority were men (53.8%), while women accounted for 46.2% of the participants. Most participants were under 30 years old (43.9%), followed by those aged 30–40 (38.2%). Out of the 212 participants, the two most reported academic qualifications were Bachelor of Medicine and Bachelor of Surgery (MBBS) (general practitioners) (56.1%), followed by board-certified family physicians (33%). Regarding the exposure of participants to migraine, approximately 21.2% of participants reported having completed a neurology internship, while 16% (34 participants) indicated that they personally suffer from migraines.

**Table 1:** Sociodemographic characteristics (*n* = 212)

Parameter	Category	*n* (%)
**Age (years)**	Less than 30	93 (43.9%)
	30 to 40	81 (38.2%)
	40 to 50	30 (14.2%)
	More than 50	8 (3.8%)
**Gender**	Male	114 (53.8%)
	Female	98 (46.2%)
**Academic qualification**	MBBS graduate	119 (56.1%)
	Board certified	70 (33.0%)
	Master’s degree	16 (7.5%)
	PhD degree	7 (3.3%)
**Professional experience (years)**	Less than 5	121 (57.1%)
	5 to 10	42 (19.8%)
	10 to 20 More than 20	36 (17.0%) 12 (6.1%)
**Completed neurology internship**	Yes	45 (21.2%)
	No	167 (78.8%)
**Suffer from migraine**	Yes	34 (16.0%)
	No	178 (84.0%)

**Figure 1:**
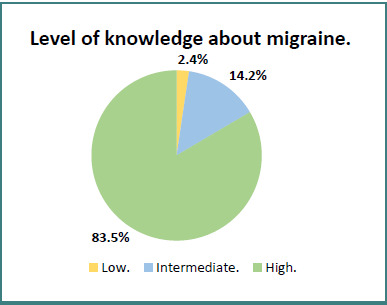
Participants' level of knowledge (based on the recognition of ICHD-3 features)

In our study sample, 177 participants (83.5%) recognized at least 6 out of the 7 diagnostics criteria of migraine without aura and were considered to have a high level of knowledge about the migraine diagnostic criteria. An intermediate level of knowledge (3–5 correct criteria) was observed in 14.2% of participants, while 2.4% had a low level of knowledge (fewer than three correct criteria). Figure 1 illustrates the distribution of knowledge levels.

Despite the overall high level of knowledge, many participants incorrectly identified atypical criteria as features of migraine. For example, some participants mistakenly classified migraine headaches as 'severe to very severe', contrary to the ICHD-3 criteria, which describe migraine pain as 'moderate to severe'. Additionally, 52.4% incorrectly associated migraines with a sudden onset. The least frequently reported atypical features were bilateral headache (14.6%) and band-like distribution (11.3%).

The participants' attitude towards migraine and its management is presented in Table 2. Overall, most participants agreed that migraines cause a high burden on the healthcare system. Most of the participating physicians considered migraine challenging to diagnose and manage; however, more than half of them (62.7%) agreed that they feel comfortable diagnosing migraine. Regarding the attitude of participants towards management, an overwhelming majority (more than 90%) agreed that lifestyle modifications are essential in the management of migraine, and more than 80% reported that migraine should be managed in primary health care facilities and only referred when it is refractory to treatment offered in the primary health care settings. Two-thirds of the participants (67.9%) disagreed with the statement suggesting that migraine is outside the primary health care scope.

**Table 2:** Participants' attitudes regarding migraine management (*n* = 212)

Item	Strongly disagree	Disagree	Neutral	Agree	Strongly agree
**Migraine headache has a high burden on the health care system**	1 (0.5%)	14 (6.6%)	49 (23.1%)	96 (45.3%)	52 (24.5%)
**Migraine headache is challenging to diagnose**	1 (0.5%)	55 (25.9%)	67 (31.6%)	68 (32.1%)	21 (9.9%)
**I feel comfortable diagnosing a migraine patient**	0 (0%)	17 (8%)	62 (29.2%)	95 (44.8%)	38 (17.9%)
**Migraine headache is challenging to manage**	1 (0%)	38 (17.9%)	58 (27.4%)	82 (38.7%)	33 (15.6%)
**Lifestyle modifications are essential in the management of all migraine patients**	0 (0%)	5 (2.4%)	13 (6.1%)	90 (42.5%)	104 49.1%)
**Migraine patients should be managed by primary care physicians and only referred if the headache is refractory**.	4 (1.9%)	16 (7.5%)	17 (8%)	88 (41.5%)	87 (41%)
**Migraine headache management is outside the scope of primary health care physicians, and patients should be referred to a specialist**.	52 (24.5%)	92 (43.4%)	35 (16.5%)	19 (9%)	14 (6.6%)

The primary management strategy among participants focused on pharmacological treatments (68%), while 22% emphasized non-pharmacological approaches, and 10% relied on referrals to neurologists. Regarding acute management, the most commonly prescribed medications were NSAIDs (50.9%), followed by triptans (34%). Almost two-thirds of the participants (63.7%) assessed treatment efficacy in the acute attack based on pain relief and/or improvement after two hours, along with relief of the most bothersome symptoms. On the other hand, the most prescribed preventive medications were beta-blockers (60.8%). More than half of the participants (56.1%) initiated preventive treatment based on the frequency of migraine attacks, and the second most common criterion was the impact on the patient's quality of daily life (27.4%). A small proportion (1.4%) did not prescribe acute treatments, and 20.3% did not prescribe preventive medications. Regarding how familiar they were with the newly approved acute and preventive medications, it was noted that the majority of the participating physicians were not familiar with the new acute medication options like ditans and geptans (62.3%) along with the new preventive medications like anti-CGRPs (70.8%). Table 3 provides the details of the participants' responses regarding the acute and preventive treatment.

**Table 3:** Participants' responses on acute and preventive migraine medications (*n* = 212)

Parameter	Category	*N* (%)
**Acute treatment**		
Treatment efficacy was assessed based on	Pain relief after 2 hours Pain improvement after 2 hours Relief of the most bothersome symptom All of the above None of the above	17 (8%) 39 (18.4%) 13 (6.1%) 135 (63.7%) 8 (3.8%)
Medications prescribed in acute attacks	Paracetamol (with or without codeine). NSAID Triptans Ergot derivatives Don’t prescribe acute medications	26 (12.5%) 108 (50.9%) 72 (34%) 3 (1.4%) 3 (1.4%)
Familiar with the newly approved acute medications (ditans and gepants)?	Lasmiditan Ubrogepant Rimegepant All of them None of them	18 (8.5%) 10 (4.7%) 14 (6.6%) 38 (17.9%) 132 (62.3%)
**Preventive treatment**		
Criteria to start preventive treatment	Patient describes migraine as severe Failure of 1^st^ line treatment Impact on daily life Frequency of attacks Other criteria	11 (5.2%) 8 (3.8%) 58 (27.4%) 119 (56.1%) 16 (7.5%)
Medications prescribed for prevention.	Beta-blocker	129 (60.8%)
	Topiramate Amitriptyline Don’t prescribe preventive medications	12 (5.7%) 28 (13.2%) 43 (20.3%)
Are you familiar with the newly approved preventive medications (CGRP monoclonal antibodies)?	Erenumab	10 (4.7%)
	Galcanezumab	5 (2.4%)
	Fremanezumab Eptinezumab All of them None of them	1 (0.5%) 11 (5.2%) 35 (16.5%) 150 (70.8%)

The participants' responses to the investigations and referral aspects are shown in Table 4. About 64.6% of participants reported not routinely ordering investigations, while 35.4% frequently did so for specific indications, such as new-onset migraine in patients over 50 years (84%), abnormal clinical examinations (73.3%), and atypical aura (52%). The most frequently ordered investigations were ophthalmic examination and evaluation (62.6%), blood tests for vitamins and minerals (58.6%), and brain imaging (54.6%). Regarding the referral patterns, the most common reason for referral was the presence of red flags (78.3%), with most physicians referring patients without red flags only after multiple failed treatment attempts. Approximately 10% of participants used referral as their primary management strategy.

**Table 4:** Participants' responses to the investigations and referral aspects (*n* = 212)

Parameter	Category	*N* (%)
Do you usually order investigations (n = 212)?	Yes	75 (35.4%)
	No	137 (64.6%)
If yes, under what condition (n = 75)?	New onset migraine above 50 years old	63 (84%)
	Abnormal clinical examination	55 (73.3%)
	Atypical aura	39 (52%)
	Throbbing pain always on the same side All migraine patients Other conditions	19 (25.3%) 10 (13.3%) 10 (13.3%)
If yes, what type of investigations (n = 75)?	Ophthalmic examination Blood tests for vitamins and minerals Brain imaging (CT/MRI) Sinus X-Ray EEG Other investigations	47 (62.6%) 44 (58.6%) 41 (54.6%) 36 (48%) 7 (9.3%) 17 (22.6%)
Reason for referral	Presence of red flags For better diagnosis Presence of therapeutic challenges Based on the patient’s request Other reasons	166 (78.3%) 21 (9.9%) 16 (7.5%) 5 (2.4%) 4 (1.9%)
When do you refer the case to a specialist?	Once seen After the failure of the 1^st^ treatment attempt Refractory cases with treatment failures I don’t usually refer migraine patients	11 (5.2%) 38 (17.9%) 155 (73.1%) 8 (3.8%)

Table 5 presents the factors associated with knowledge levels among participants. Significant associations were observed with age and gender. Participants under 30 demonstrated the highest knowledge levels (mean score: 6.34 ± 0.715), while those aged 40–50 had the lowest (mean score: 5.37 ± 2.189; *P* = 0.002). Also, women had significantly higher knowledge levels (6.23 ± 0.939) compared to men (5.89 ± 1.441) (*P* = 0.041). Other factors, including academic qualifications, years of experience, completion of a neurology internship, personal history of migraines, predominant management strategies, and ordering of investigations, showed no significant association with knowledge levels.

**Table 5:** Factors associated with participants' knowledge about migraine

Parameter	Migraine knowledge score (Mean ± SD)	T/F Value	*P* value
**Age (years)** Less than 30 (*n* = 93) 30 to 40 (*n* = 81) 40 to 50 (*n* = 30) More than 50 (*n* = 8)	6.34 ± 0.715 5.98 ± 1.140 5.37 ± 2.189 6.00 ± 1.414	5.138	0.002*
**Gender** Male (*n* = 114) Female (*n* = 98)	5.89 ± 1.441 6.23 ± 0.939	-2.060	0.041*
**Academic qualification** MBBS graduate (*n* = 119) Board certified (*n* = 70) Master’s degree (*n* = 16) PhD degree (*n* = 7)	6.08 ± 1.283 6.10 ± 1.181 5.69 ± 1.401 6.00 ± 0.816	0.508	0.667
**Professional experience (years)** Less than 5 (*n* = 121) 5 to 10 (*n* = 42) 10 to 20 (*n* = 36) More than 20 (*n* = 12)	6.17 ± 1.070 5.93 ± 1.438 5.78 ± 1.495 6.08 ± 1.320	1.110	0.346
**Completed neurology internship** Yes (*n* = 45) No (*n* = 167)	5.71 ± 1.487 6.14 ± 1.158	1.810	0.075
**Suffer from migraine** Yes (*n* = 34) No *n* = 178)	6.06 ± 1.229 6.05 ± 1.250	-0.035	0.972
**Order additional investigations** Yes (*n* = 75) No (*n* = 137)	6.04 ± 1.179 6.06 ± 1.282	0.105	0.916
**Type of management** Mainly non-pharmacological (*n* = 47) Mainly pharmacological (*n* = 143) Tend to refer to a specialist (*n* = 22)	6.28 ± 0.902 6.05 ± 1.286 5.59 ± 1.501	2.307	0.102

## DISCUSSION

This was the first cross-sectional study to evaluate the level of knowledge, attitude, and practice of migraine management among primary healthcare physicians in the city of Al-Madinah Al-Munawarah, Saudi Arabia. The Scientific Research Ethics Committee of Taibah University, Saudi Arabia, granted ethical approval. The researchers distributed a validated questionnaire. The study included 212 primary healthcare physicians who volunteered and agreed to complete the questionnaire, surpassing our initial target sample size.

Regarding the sociodemographic data of the participants, the majority were under the age of 50 (> 95%) with a male-to-female ratio of roughly 1.16:1. More than half of our sample were assigned as general practitioners, and almost a third were officially family physicians. The rest of the participants completed their Master’s or PhD degrees. The majority (76.9%) had a professional experience of less than 10 years. Only 16% of our sample reported having migraines.

We evaluated the level of knowledge regarding the diagnosis of migraines based on the ICHD-3 criteria and found that most participants (83.5%) correctly identified at least 6 out of 7 diagnostic elements. This level of knowledge was comparable to findings from a local study conducted in Jeddah, where over 80% of primary healthcare providers accurately recognized most diagnostic criteria. However, in our study, we observed that younger age (< 30 years old) and female gender were significantly correlated with a higher level of knowledge. Conversely, young physicians (23-35 years) and those with an experience of ≤ 5 years had a lower percentage of adequate knowledge in the Jeddah study [16].

There is significant variation in migraine knowledge among general physicians across different countries. A cross-sectional study conducted in Burkina Faso revealed that 80.2% of general practitioners understood the diagnostic criteria outlined in the ICHD-3 [15]. In contrast, only 10.5% of the sample in a study by Murat *et al*. in Turkey could identify every migraine diagnostic criterion, although over 60% of the sample had up to 10 years of experience as primary healthcare physicians [12]. In another regional study in Spain, general practitioners (GPs) were asked to diagnose and treat factitious clinical patients who met all ICHD-3 criteria. More than 50% of GPs failed to diagnose migraine without aura correctly and rather employed the diagnosis of tension-type headache or mixed headache [20].

In our study, the most frequently ordered investigations for migraine were ophthalmic examination and evaluation, blood tests for vitamins and minerals, and brain imaging. These were predominantly ordered for cases of new-onset migraines in patients over 50 years of age or those presenting with abnormal clinical examinations. Similarly, a study by Minen *et al*. reported that MRI was the most frequently ordered investigation for a new onset headache or headache with neurological symptoms [21]. Consistent findings were also observed in two other studies [15,22]. In contrast, a study conducted in Cameroon revealed that approximately one-third of participants believed brain imaging was necessary for diagnosing headaches in general [23]. This was comparable to findings from a study conducted in Turkey, where a significant portion of participants also relied on imaging for headache diagnosis [12].

In our study, only 62.7% of participants reported feeling comfortable diagnosing migraines, which is notably lower than findings in other studies. For instance, Minen *et al*. reported that 98.7% of participants were confident in diagnosing migraines [21], and another study found that 80% were comfortable with the diagnosis [22].

Regarding attitudes toward migraine management, more than 90% think that lifestyle modifications are essential, which is significantly higher compared to a study by Mehorta *et al*., where only 25% focused on non-pharmacological treatment [24].

In terms of acute migraine management, the majority of participants (68.9%) primarily focused on pharmacological treatments, with NSAIDs being the most prescribed medication (50.9%), followed by triptan (30.7%) and paracetamol (12.9%). These findings align with the recommended stepwise approach for the treatment of acute migraine attacks [25]. The International Headache Society (IHS) recommended that the outcome measure of treatment efficacy should be a sustained pain-free response within 2 hours [26]. Consistent with this guideline, 63.7% of our participants assessed treatment success based on either pain relief, resolution of bothersome symptoms, or improvement within 2 hours.

The recommendations regarding the indications of initiating chronic preventive therapy are based on the frequency of attacks and the impairment of daily life [27]. Our study found that over half of the participants (56.1%) adhered to these guidelines, using the frequency of migraine attacks as the primary reason for prescribing preventive medications. In contrast, 27.4% of primary healthcare providers relied on the impact of migraine attacks on patient’s quality of life. Regarding the agents used in prophylactic treatment, 60.8% would prescribe beta-blockers for chronic migraine patients, whereas 13.2% would prefer amitriptyline. One-fifth of the participants (20.3%) chose not to prescribe preventive medications to their patients. The American Headache Society (AHA) and American Academy of Neurology (ANA) guidelines recommend beta-blockers and topiramate as effective preventive therapy for chronic migraine [28]. When asked about referrals, our survey revealed that 78.3% of PHC physicians would refer patients with red flags. Other reasons were for the sake of better diagnosis (9.9%), cases with therapeutic challenges (7.5%), or based on patient request (2.4%). When assessed about the newly approved medications, most participants were unfamiliar with either new abortive or preventive medications (e.g., ditans, gepants, or CGRP monoclonal antibodies). We did not find any clinical studies that evaluated primary healthcare physicians’ knowledge concerning novel FDA-approved migraine drugs. This result highlights a lack of awareness and adoption of emerging trends in migraine management among primary healthcare providers.

In the Jeddah study, the initial management of chronic migraine often focused on lifestyle modifications and trigger management, with 96.3% of general practitioners (GPs) adopting this approach. In addition, 86.8% provided acute and preventive management for their patients. Beta-blockers, angiotensin receptor blockers, and tricyclic antidepressants were reported as effective preventive therapy by 69.1% of participants [16].

The practice of migraine management varied significantly across different regions. In the USA, an online survey showed that over 70% prescribed NSAIDs as an abortive therapy. Acetaminophen was the second most common drug (63.2%) [29]. In Burkina Faso, triptan was only given by 2.6%, whereas paracetamol and NSAIDs were chosen by 48.7% and 40%, respectively. The most common prophylaxis therapy was offered, including amitriptyline (27.8%), ergot derivative (18.9%), NSAIDs (16.7%), topiramate (13.3%), and triptan (12.2%). Concerning referral patterns, better treatment was the main reason for referral among most participants [15].

All GPs in the Spain study prescribed NSAIDs in acute management, and 63.8% recommended using triptan as an adjunct option. Additionally, 71.4% of GPs indicated they may offer preventive treatment if necessary. Beta-blockers were the most chosen medication (62.8%), followed by amitriptyline (21.9%) [20].

In a European study based on five countries (France, Italy, Germany, Spain, and the UK), 82% of GPs reported that they would treat chronic migraine patients without referring them to a specialist. The prescription of preventive treatment was opted by 72% of participants [30].

Despite our participants' satisfactory knowledge and practices, educational campaigns targeting general practitioners and primary healthcare providers are crucial for a deeper grasp of clinical aspects, particularly newly approved therapeutic approaches. Several studies employed educational programs that were specifically designed for primary healthcare physicians. The post-program results revealed a significant improvement in participants' knowledge, attitude, and practice regarding migraine [23,31,32].

It is worth noting that our study—despite being the first study addressing this specific topic in Al-Madinah Al-Munawarah, KSA—has some limitations that should be addressed in similar subsequent studies in the region. Firstly, we adopted a convenient sampling technique that could not guarantee the randomness of the sample selection. Our results and observations, although helpful for guiding the efforts in assessing and improving the management of migraine locally, cannot be confidently generalized to the whole population of KSA or the international population. Also, our study was primarily descriptive, and we recommend advanced analytical studies to assess this particular topic for a deeper look. Finally, it would be more suitable to use a locally validated questionnaire, if applicable and available, to minimize the potential limitations of a previously validated questionnaire in different regions.

## CONCLUSION

The knowledge of migraine headaches among primary healthcare physicians in Al-Madinah Al-Munawarah was generally good, with 83.5% of participants demonstrating good knowledge according to ICHD-3 diagnostic criteria. Factors associated with a higher level of knowledge were female gender and age group under 30 years. Regarding attitudes toward migraine management, 68% of participants emphasized pharmacological approaches, with NSAIDs being the most commonly prescribed treatment for acute attacks and beta-blockers as the preferred prophylactic agents. Surprisingly, most participating physicians were unfamiliar with the new acute medication options and preventive medications.

The findings of this study demonstrate good knowledge, attitudes, and practice habits among the participants. However, significant gaps were identified in their awareness of recent advances in migraine treatment. We recommend implementing continuous education programs focused on the latest developments in migraine management for primary care physicians in Al-Madinah Al-Munawarah.

## Personal thanks

The authors thank the primary care physicians whose participation made this study possible.
